# Editorial Comment: Penile cancer information on the internet: a needle in a haystack

**DOI:** 10.1590/S1677-5538.IBJU.2020.06.04

**Published:** 2020-09-02

**Authors:** Gustavo Cardoso Guimarães

**Affiliations:** 1 Departamento de Oncologia Cirúrgica Beneficência Portuguesa de São Paulo São PauloSP Brasil Chefe do Departamento de Oncologia Cirúrgica, Beneficência Portuguesa de São Paulo, São Paulo, SP, Brasil

Jiasian Teh ^1, 2, 3^, Stephanie Op’t Hoog ^1, 2^, Tatenda Nzenza ^1, 2, 3^, Catriona Duncan ^1, 2, 4^, Judy Wang ^1, 5^, Matija Radojcic ^1^, Cheng Feng ^1^, Nathan Lawrentschuk ^1, 3, 5^

^1^ Department of Surgery, Austin Hospital, University of Melbourne, Melbourne, Vic., Australia; ^2^ Young Urology Researchers Organization, Melbourne, Vic., Australia; ^3^ Department of Surgical Oncology, Peter MacCallum Centre, Melbourne, Vic., Australia; ^4^ North East Urology, Melbourne, Vic., Australia; 5 Olivia Newton-John Cancer Research Institute, Austin Hospital, Melbourne, Vic., Australia.

BJU Int. 2018 Nov;122 Suppl 5:22-26.

**DOI: 10.1111/bju.14532 | ACCESS: 10.1111/bju.14532**

## COMMENT

In this interesting paper, Dr Jiasian Teh and colleagues at Austin Hospital from University of Melbourne, raise an important theme these days. The quality of health information on the internet, especially in penile cancer. Where one of the main factors related to delay in seeking help is due to misinformation ( 1 ).

We have recently witnessed a large number of fake news in many areas, including health, leading people to fear or distrust treatments and vaccines ( 2 ).

In the analysis, the authors compared the quality of information about penile cancer on the internet and to compare the quality of information from developed countries with developing countries.

They assess 750 websites in English, French, German, Spanish and Portuguese. And two independent examiners analyzed the first 150 in each language, using the key word ‘penile cancer’ in all languages.

The Health on the Net (HON) principles were applied to websites using the Google search engine imbedded with HON toolbar, and the examiners search for HON accredited websites.

Of the 750 websites analysed, 10.4% were HON accredited. There were significantly more HON accredited websites in English and French compared with Portuguese (P = 0.009 and P = 0.0007). A total of 45% of websites were sponsored by Commercial enterprise and 27% were sponsored by Government organisations.

The authors concluded that there is A lack of validation of penile cancer internet resources should be appreciated by clinicians. And more importante in our opinions is that there is a discrepancy in the quality of websites between languages, with significantly more resources available in the developed world. Limited available web resources in Spanish and Portuguese contribute to disparities in information access and disease outcomes.

So, we should work to improve the quality of medical information in our language and combat fake news.
